# A Rare Case of Wirsung Duct Duplication with Chronic Pancreatitis

**DOI:** 10.5334/jbsr.3447

**Published:** 2024-03-20

**Authors:** Marie Hollans, Jean-Luc Engelholm

**Affiliations:** 1 Department of Radiology, Joseph Bracops Hospital, Université Libre de Bruxelles; 2 Department of Radiology, Joseph Bracops Hospital, Université Libre de Bruxelles

**Keywords:** chronic pancreatitis, pancreas divisum, magnetic resonance cholangiopancreatography, fish-tail pancreas

## Abstract

*Teaching point:* Wirsung duct duplication is a very rare condition that can lead to chronic pancreatitis.

## Case History

A 68-year-old male patient with a history of alcoholism was referred to the gastroenterologist for altered general status associated with bowel disorders.

His lab workup showed disturbed liver function. Upper and lower gastrointestinal endoscopies were negative.

**Figure 1:**
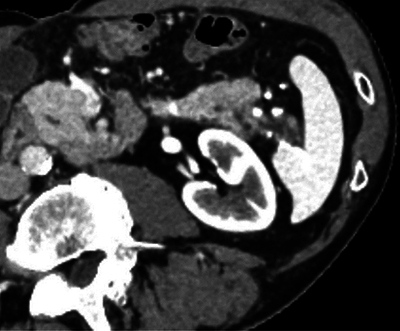
CT scan in arterial phase, showing caudal division of the tail of the pancreas and chronic pancreatitis changes posteriorly.

**Figure 2:**
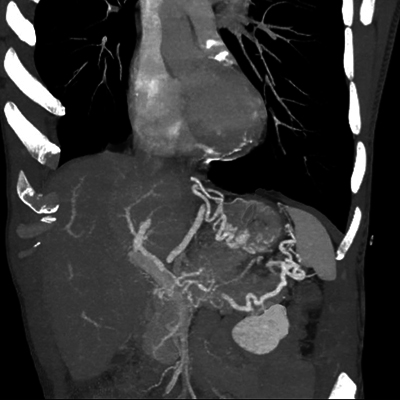
CT performed during portal phase, Maximum Intensity Projection 3D-reconstruction showing splenic vein thrombosis with subsequent gastro-epiploic and fundus varices.

An abdominal computed tomography (CT) scan with portal phase was performed, showing dilatation of the caudal pancreatic duct (Figure 1) along with splenic vein thrombosis and gastro-epiploic and fundus varices (Figure 2).

**Figure 3:**
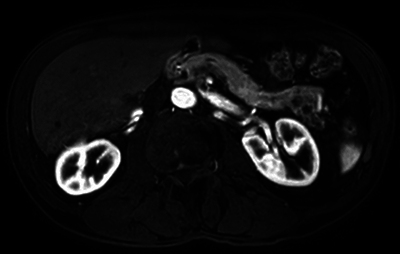
Axial cut in T1 DIXON sequence in arterial phase showing caudal division of the tail of the pancreas and chronic pancreatitis changes.

**Figure 4:**
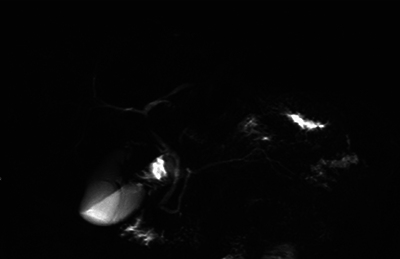
MR cholangiopancreatography, RARE sequence showing caudal division of the Wirsung duct and chronic pancreatitis changes.

Magnetic resonance cholangiopancreatography showed a rare anatomical variant with duplication of the Wirsung duct along with a bifid appearance of the distal portion of the pancreas (Figures 3 and 4) and ductal alterations due to chronic pancreatitis were noted in the posterior tail.

## Comment

Chronic pancreatitis is a pathology most often encountered in cases of alcohol abuse or recurrent biliary pancreatitis. Rarely, some congenital anatomical variants may be complicated by chronic pancreatitis, although such variants most often remain asymptomatic [1]. Among the most common, pancreas divisum consists of non-fusion between the ventral and dorsal portions and is an incidental finding in the majority of cases. However, this anomaly is more frequently observed in patients presenting with chronic abdominal pain and idiopathic pancreatitis [1]. The CT and magnetic resonance imaging (MRI) images in this case illustrate a rare pancreatic malformation consisting of a division of the Wirsung duct accompanied by alterations from chronic pancreatitis in the “posterior tail” in a patient with persisting abdominal pain and alcohol abuse. Awareness and recognition of pancreatic malformations are crucial for the therapeutic management of pancreatitis.
